# A novel circular RNA circRBMS3 regulates proliferation and metastasis of osteosarcoma by targeting miR-424-eIF4B/YRDC axis

**DOI:** 10.18632/aging.204567

**Published:** 2023-03-09

**Authors:** Zhe Gong, Panyang Shen, Haitao Wang, Jinjin Zhu, Kaiyu Liang, Kefan Wang, Yunfeng Mi, Shuying Shen, Xiangqian Fang, Gang Liu

**Affiliations:** 1 Department of Orthopaedic Surgery, Sir Run Run Shaw Hospital, Medical College of Zhejiang University and Key Laboratory of Musculoskeletal System Degeneration and Regeneration Translational Research of Zhejiang Province Sir Run Run Shaw Institute of Clinical Medicine of Zhejiang University, Hangzhou 310016, Zhejiang Province, China; 2 Department of Orthopaedic Surgery, Ningbo First Hospital, Ningbo 315010, China

**Keywords:** circRNA, osteosarcoma

## Abstract

Circular RNAs (circRNAs) have been demonstrated to have critical regulatory roles in tumorigenesis. However, the contribution of circRNAs to OS (osteosarcoma) remains largely unknown. circRNA deep sequencing was performed to the expression of circRNAs between OS and chondroma tissues. The regulatory and functional role of circRBMS3 (a circRNA derived from exons 7 to 10 of the *RBMS3* gene, hsa_circ_0064644) upregulation was examined in OS and was validated *in vitro* and *in vivo*, upstream regulator and downstream target of circRBMS3 were both explored. RNA pull down, a luciferase reporter assay, biotin-coupled microRNA capture and fluorescence *in situ* hybridization were used to evaluate the interaction between circRBMS3 and micro (mi)-R-424-5p. For *in vivo* tumorigenesis experiments, Subcutaneous and Orthotopic xenograft OS mouse models were built. Expression of circRBMS3 was higher in OS tissues due to the regulation of adenosine deaminase 1-acting on RNA (ADAR1), an abundant RNA editing enzyme. Our *in vitro* data indicated that ShcircRBMS3 inhibits the proliferation and migration of osteosarcoma cells. Mechanistically, we showed that circRBMS3 could regulate *eIF4B* and *YRDC*, through ‘sponging’ miR-424-5p. Furthermore, knockdown of circRBMS3 inhibited malignant phenotypes and bone destruction of OS *in vivo*. Our results reveal an important role for a novel circRBMS3 in the growth and metastasis of malignant tumor cells and offer a fresh perspective on circRNAs in OS progression.

## INTRODUCTION

Osteosarcoma (OS) has a high incidence in the adolescent population, among whom it is the most common primary malignant bone sarcoma [1]. Recently, there has been a significant improvement in the prognosis of OS patients owing to the combination of neoadjuvant chemotherapy, advanced diagnostic methods, and surgery [2]. Despite such progress, many patients still respond poorly to normative drug therapy and curative tumor resection and suffer local relapse or distant metastasis [3]. Moreover, little is known concerning the underlying mechanisms surrounding oncogenesis, development, distant metastasis, and drug resistance in OS. Indeed, no precise diagnostic markers or effective therapeutic targets for OS have been discovered, resulting in a diagnostic and treatment “bottleneck.”. Therefore, it is crucial to investigate innovative strategies to improve clinical outcomes.

Competing endogenous RNAs (ceRNAs) are critical in determining gene expression regulation in many malignant tumors. Dysregulation and disruption of ceRNA networks have been found to may be predominant in tumorigenicity [4]. Circular RNAs (CircRNAs), which are remarkably stable, show strong evolutionary conservation and high abundance and are crucial in regulating gene expression by mimicking ceRNAs. Intracellular circRNAs may act as miRNA ‘sponges’, sequestering miRNA when it binds with MREs (microRNA response elements), leading to strong inhibition of miRNA activity and, therefore, gene expression regulation [5]. Several studies have reported the circRNA role as “miRNA sponges”, among which CDR1 as or ciRS-7 [6, 7] appears to be the most understood. Containing 74 canonical binding sites for miR-7, CDR1as effectively combines with and sponges miR-7, leading to the attenuated interaction between CDR1 and miR7. circRNA dysregulation has been found in various tumors, and a previous study indicated that certain circRNAs are aberrantly expressed in OS [8]. Although a few preliminary investigations have been conducted regarding the circRNAs role in OS [9–11], the circ-RNAs overall pathophysiological contributions to OS are still non-understood to a large extent.

microRNAs (miRNAs) are 19–25 nucleotides long, non-coding RNAs that directly govern target gene expression by binding to the 3’-untranslated position of target mRNAs. miRNAs modulate various pathological processes, including OS progression [12]. Aberrant miRNA expression is suggested to be frequent in various tumors, exerting several impacts to promote and/or inhibit tumorigenesis, development, and metastasis [13, 14]. An association between the MiR-424-5p (miR-424) dysregulation and many malignant tumors has been found [15, 16]. However, how circRNA regulates miR-424 in OS is still poorly understood.

Herein, we used RNA-sequencing (RNA-seq) to compare the circRNAs expression between OS and chondroma tissues. We show that a circRNA (circRBMS3) originating from the *RBMS3* gene transcript is upregulated in OS tissue. The circRBMS3 role in the growth and metastasis of tumors needed to be further explored.

## RESULTS

### circRNA expression patterns in human OS and chondroma tissues

**Figure 1:**
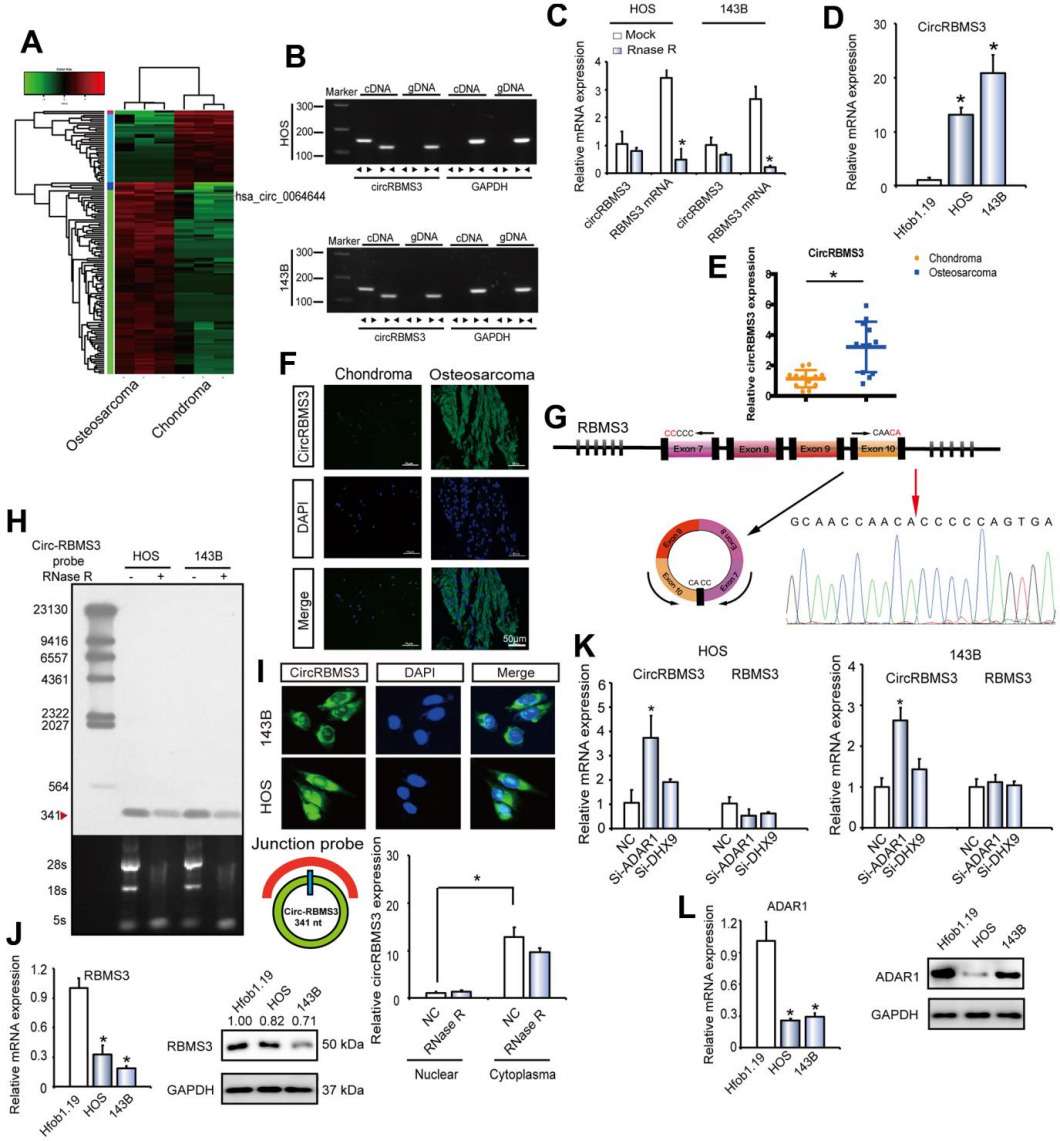
**circRBMS3 validation and expression in OS tissue and cells.** (**A**) Heat map of all differentially expressed circRNAs between chondroma and OS tumor tissues. (**B**) The presence of circRBMS3 was validated in HOS and 143B OS cell lines by RT–qPCR. Divergent primers amplified circRBMS3 in cDNA, but not in genomic DNA. *GAPDH* was used as a negative control. (**C**) The expression of circRBMS3 and *RBMS3* mRNA in HOS and 143B cells treated with or without RNase R was detected by qPCR. The relative levels of circRBMS3 and RBMS3 mRNA were normalized to the value measured in the mock treatment. Data represent the mean ± SD (n = 3). * *P* < 0.05. (**D**) CircRBMS3 expression in hFOB1.19 and OS (OS) cell lines (HOS and 143B) was evaluated by RT-qPCR. Data represent the mean ± standard deviation (SD) (n = 3). * *P* < 0.05 (**E**) CircRBMS3 expression was higher in human OS than in chondroma tissue. Data represent the mean ± SD (n = 12). * P < 0.05. (**F**) CircRBMS3 expression was higher in human OS than in chondroma tissue. Representative images are shown (400 × magnification). (**G**) Schematic illustration showing *RBMS3* exon 7–10 circularization forming circRBMS3 (black arrow). The presence of circRBMS3 was validated by RT–qPCR, followed by Sanger sequencing. Red arrow represents “head-to-tail” circRBMS3 splicing sites. (**H**) Northern blots for detecting circRBMS3 in HOS and 143B cells treated with or without RNase R digestion. The upper panels show the probed blots of circRBMS3, and the red triangle represents the circRBMS3 band size (341 bp). The lower panels show the gel electrophoretic results of RNA with or without RNase R digestion. (**I**) RNA fluorescence *in situ* hybridization (FISH) showed that circRBMS3 was predominantly localized in the cytoplasm. CircRBMS3 probes were labeled with Alexa Fluor 488. Nuclei were stained with DAPI. Scale bar, 50 μm. Upper panel: FISH with junction-specific probes indicates the cellular localization of circRBMS3. Scale bars = 5 μM. Lower panel: circRBMS3 was detected in different cell fractions. Nuclear and cytoplasmic RNA was extracted, and junction primers were used for circRBMS3 detection. U6 was used as an internal control of nuclear RNA, and *GAPDH* was used as internal control for cytoplasmic RNA. Values are the average ± SD of 3 independent experiments. (**J**) *RBMS3* expression in hFOB1.19 and OS (OS) cell lines (HOS and 143B) was evaluated by WB and RT-qPCR. Data represent the mean ± standard deviation (SD) (n = 3). * *P* < 0.05. (**K**) RT-qPCR for *RBMS3* mRNA and circRBMS3 upon *DHX9* and *ADAR1* depletion using RNAi in OS cell lines. (**L**) *ADAR1* expression in hFOB1.19 and OS cell lines (HOS and 143B) was evaluated by RT-qPCR and WB. Data represent the mean ± standard deviation (SD) (n = 3). * P < 0.05.

From clinical OS and chondroma tissues, we performed circRNA deep sequencing of ribosomal RNA-depleted total RNA for generating a circRNA-profiling database. The RNA sequencing from three human osteoblastic OS samples and three chondroma tissues was performed by an Illumina Hiseq X Ten. The mapping of the obtained reads was to a ribosomal RNA reference (Bowtie2, http://bowtie-bio.sourceforge.net/bowtie2/) as well as to a reference genome (TopHat2, http://ccb.jhu.edu/software/tophat/) [17, 18]. The extraction and alignment of twenty bases from either end of the unmapped reads were to the reference genome in order to identify distinct anchor positions inside the splice sites. The alignment Anchor reads in the reverse orientation (head-to-tail) demonstrated circRNA splicing and were subsequently submitted to find_circ (https://omictools.com/find-circ-tool) for the circRNAs identification [19]. A candidate circRNA was one that was detected if at minimum one of two distinctive back-spliced reads from a single sample. Identification of 25224 circRNAs was made using this method (Supplementary Figure 1A) [20]. The annotation of the detected candidates was then performed via the RefSeq database [21]. The origination of most circRNAs was from protein-coding exons, whereas other originated from introns, 5’-UTRs, and 3’-UTRs (Supplementary Figure 1B). The majority of detected circRNAs did not exceed 2000 nucleotides (nt) in length (Supplementary Figure 1C). There was a non-significant variation in the chromosomal distribution of detected circRNAs between the OS and chondroma group, whereas there was a downregulation in the circRNAs total expression in the OS group (Supplementary Figure 1D, 1E). Analysis of circRNAs numbers from host genes demonstrated the ability of one gene for multiple circRNAs productions (Supplementary Figure 1F), which accords with prior findings [22]. The circRNAs abundance within a single gene locus (n¼3,687) was investigated. The thick line indicates the median, while the ends of the boxes represent the 25th and 75th centiles, as well as the bars represent the 5th and 95th centiles (Supplementary Figure 1G). This finding indicated the frequently most abundantly expressed circRNA isoform from one gene location. The OS and chondroma groups displayed differential circRNA expression patterns (Figure 1A). Next, the further selection was performed. The identification of the 214 circRNAs differentially expressed in OS and chondroma was performed by RNA-seq mapping to the reference genome (hg38, human genome), with | log2FC(OS/chondroma) | >1, among which 183 circRNAs showed significant overexpression and 31 circRNAs showed significant downregulation. Then, we listed the top 15 significantly upregulated circRNAs in OS and chondroma with log2FC(OS/chondroma) >1 and mean chondroma value > 0 (Supplementary Table 1). We focused on candidates with the most significant differential expression between OS and chondroma groups, then matched them with circbase (http://www.circbase.org/. Among these specific candidates, novel_circ_0064644, listed in the top three upregulated circRNAs in OS, formed by exon 7 circularizing to exon 10 of *RBMS3*, attracted our attention.

### Identification of circRBMS3 as a circRNA

To verify that exons 7 to 10 of *RBMS3* form an endogenous circRNA, convergent as well as divergent primers were constructed to specifically amplify the *RBMS3* canonical or back-spliced forms (Figure 1B). Employing cDNA and genomic DNA (gDNA) as templates from HOS and 143B cell lines, only by using divergent primers was possible to make the circRBMS3 amplification, and there was no sign of an amplification product coming from the gDNA (Figure 1B). Performing RT-qPCR further confirmed the circRBMS3 resistance to RNase R, while *RBMS3* mRNA levels showed significant reduction following RNase R treatment (Figure 1C).

To find out the circRBMS3 role in OS development, the circRBMS3 expression level was assessed in 12 pairs of chondroma and OS tissues, respectively, as well as in OS cell lines. Taking advantage of RT-qPCR and chromogenic *in situ* hybridization (CISH) and as consistent with the RNA-seq analysis, we observed an elevated circRBMS3 expression in OS than in chondroma tissues or normal cells (Figure 1D–1F). We then performed Sanger sequencing to confirm the circRBMS3 junction (Figure 1G). Endogenous circRBMS3 was further revealed by a junction-specific probe in northern blotting (Figure 1H). To establish the circRBMS3 cellular localization, we performed fluorescence *in situ* hybridization (FISH) analysis. The junction probe revealed high cytoplasmic circRBMS3 expression in HOS cells. Additionally, qPCR analysis from different cell fractions verified the circRBMS3 predominant localization within the cytoplasm (Figure 1I, lower panel).

### The expression of circRBMS3 in OS can be regulated by ADAR1

**Figure 2:**
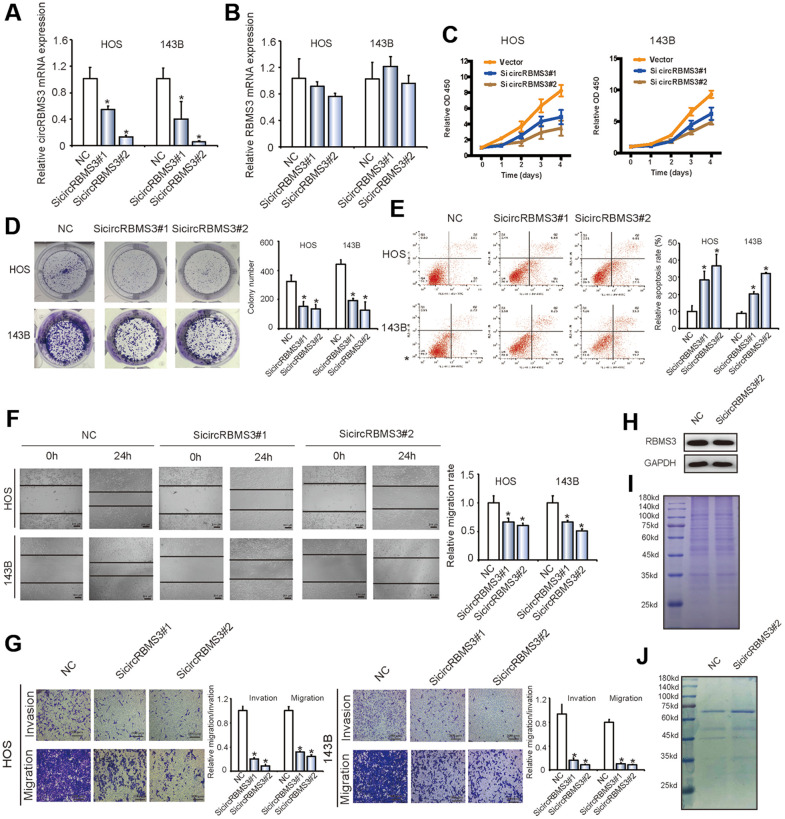
**Knockdown of circRBMS3 inhibits the migration and invasion of OS cell lines *in vitro*.** (**A**, **B**) The expression levels of circRBMS3 and *RBMS3* mRNA in HOS and 143B cells after stable transfection of circRBMS3 short hairpin RNAs or vector plasmids were detected by qPCR. Data represent the mean ± SD (n = 3). *P < 0.05. (**C**) SiRNA-mediated circRBMS3 knockdown suppressed OS cell proliferation, as determined by CCK-8 assays. Data represent the mean ± SD (n = 6). (**D**) CircRBMS3 knockdown suppresses cell growth, as determined by colony formation assays (details are shown in the insets). Error bars represent the mean ± SD of three independent experiments. * *P* < 0.05. (**E**) HOS and 143B cells were transfected with sicircRBMS3, followed by Annexin V-FITC/PI staining. The percentage of apoptotic cells is shown as the mean ± SD from three independent experiments. * *P* < 0.05, significantly different compared with the vector group. (**F**) The effect of sicircRBMS3 on cell migration capability was evaluated by wound healing assays using HOS and 143B cells. Data are the mean ± SD, n = 3. *P < 0.05. Scale bar, 200 μm. (**G**) CircRBMS3 knockdown suppresses cell migration and invasion abilities of HOS and 143B cells, as evaluated by transwell migration and Matrigel™ invasion assays. Data represent the mean ± SD (n = 3). * *P* < 0.05. Scale bar, 200 μm. (**H**) CircRBMS3 knockdown did not affect linear *RBMS3* expression. (**I**) Total cell lysates were separated by SDS-PAGE and Coomassie blue staining. (**J**) Cell lysates were precipitated with anti-RBMS3 antibody followed by SDS-PAGE and Coomassie blue staining. Transfection with circRBMS3 siRNA did not affect the interaction of *RBMS3* with its partners.

*RBMS3* is a novel candidate gene for suppressing tumors as in esophageal squamous cell carcinoma (ESCC) [23], breast cancer [24], nasopharyngeal cancer (NPC) [25], and lung squamous cell carcinoma (LSCC) [26]. Although the significant overexpression of circRBMS3 in OS cell lines, the RBMS3 protein levels were downregulated (Figure 2I), suggesting that the elevated circRBMS3 expression in OS is not simply a by-product of splicing but may be functional.

Next, we explored the reasons for circRBMS3 upregulation in OS, alongside RBMS3 mRNA and RBMS3 protein downregulation (Figure 1J). As RNA-binding proteins could govern circRNAs [27] post-transcriptionally, we suggested that circRBMS3, but not RBMS3 mRNA, is governed by specific RNA-binding proteins post-transcriptionally during human OS development. For validating our hypothesis, the circRBMS3 expression was measured in OS cell lines following the individual knockdown of all two human RNA-binding proteins [28–30] reported governing the circRNAs biogenesis broadly, including adenosine deaminase 1 acting on RNA (ADAR1) and DExH-Box Helicase 9 (DHX9). Among these two RNA-binding proteins, DHX9 is essential in OS development [31–33]. After knocking down ADAR1(p110) but not DHX9, circRBMS3 was upregulated, while RBMS3 mRNA did not show significant changes (Figure 1K). Notably, mRNA and protein levels of ADAR1 showed downregulation in OS cell lines (Figure 1L). Together, the downregulation of ADAR1 in human OS is at least partially responsible for the overexpression of circRBMS3.

### Effects of circRBMS3 on OS cell lines

For further exploration of the circRBMS3 biological functions, we introduced two circRBMS3-knockdown short interfering (si)-RNAs to target the circRBMS3 junction sites into HOS and 143B OS cells. The circRBMS3 expression showed significant downregulation in siRNA-transfected cells (Figure 2A). Meanwhile, the *RBMS3* mRNA expression showed no significant change (Figure 2B). The capabilities of HOS and 143B cells to proliferate and form colonies decreased upon circRBMS3 inhibition (Figure 2C, 2D). The influence of circRBMS3 knockdown on OS cells apoptosis rate was determined via the Flow cytometric analysis. Notably, inhibition of circRBMS3 augmented cellular apoptosis (Figure 2E).

Furthermore, circRBMS3 knockdown in HOS and 143B cells led to overexpression of Bax and cleaved-caspase3, while the downregulation of Bcl2 was indicated by western blot (Supplementary Figure 2A). Consistent with this, a wound-healing assay revealed significant suppression of cell migration in HOS and 143B cells following circRBMS3 knockdown (Figure 2F). Moreover, inhibition of circRBMS3 also led to the downregulation of migration and invasion of OS cell lines in transwell migration and Matrigel invasion assays (Figure 2G). CircRBMS3 knockdown in HOS and 143B cells also decreased E-cadherein, N-cadherein, vemitin, snail, and slug expression indicated by western blot (Supplementary Figure 2B). We also found that transfecting circRBMS3 siRNA did not affect the RBMS3 protein level (Figure 2H). Knockdown of circRBMS3 did not alter total protein levels (Figure 2I) or the interaction of RBMS3 with its partners (Figure 2J). The above findings revealed the circRBMS3 involvement in growth, migration, and invasion of the tumor cells *in vitro*.

### Molecular mechanism of circRBMS3 in OS

**Figure 3:**
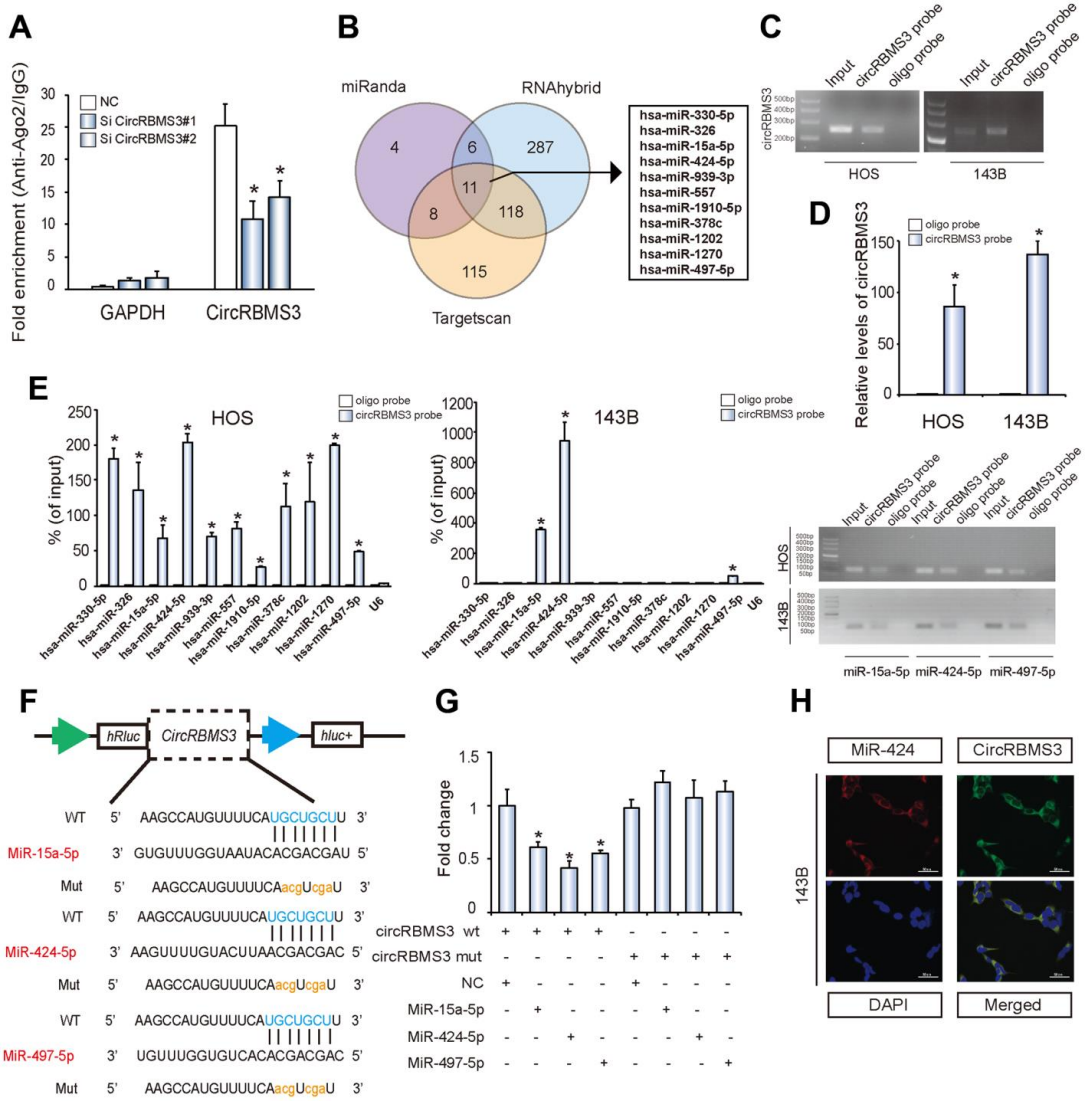
**circRBMS3 serves as a sponge for miR-424 in OS cells.** (**A**) *AGO2* RNA immunoprecipitation assay for circRBMS3 levels in 143B cells stably expressing shcircRBMS3. Data represent the mean ± SD for three experiments. * *P* < 0.05. (**B**) Schematic illustration showing overlapping of the target miRNAs of circRBMS3 predicted by miRanda, Targetscan, and RNAhybrid. (**C**, **D**) Lysates prepared from HOS and 143B cells were subjected to an RNA pull-down assay and tested by (**C**) RT–qPCR and (**D**) qPCR. Relative levels of circRBMS3 were normalized to input. Data represent the mean ± SD (n = 3). * *P* < 0.05 versus oligo probe (Student’s *t*-test). (**E**) The relative levels of 11 miRNA candidates in HOS and 143B cell lysates, as detected by qPCR and RT–qPCR. (**F**) Schematic illustration demonstrating complementary miR seed sequence with circRBMS3. Lowercase letters indicate mutated nucleotides. (**G**) 293T cells were co-transfected with miR mimics and a luciferase reporter construct containing wild-type (WT) or mutated (MUT) circRBMS3. Data represent the mean ± SD (n = 3). * *P* < 0.05. (**H**) FISH images showing co-localization of circRBMS3 and miR-424 in 143B cells. CircRBMS3 probes were labeled with Alexa Fluor 488. Locked nucleic acid miR-424 probes were labeled with Cy3. Nuclei were stained with DAPI. Scale bar, 50 μm.

CircRNA acts as a sponge for miRNA in malignant cells [6, 7]. Depending on freely available AGO2 immunoprecipitation data, such as high-throughput sequencing from doRiNA, we carried out an analysis based on the circRBMS3 abundance in the cytoplasm. We observed a great level of AGO2 occupancy in the circRBMS3 region, which is substantially conserved in many vertebrate species (Supplementary Figure 3A). for the verification of this, an RNA immunoprecipitation for AGO2 in 143B cells was performed, revealing that endogenous circRBMS3 pulled-down from AGO2 antibodies was specifically enhanced by RT-qPCR analysis in contrast with circRBMS3 knockdown cells (Figure 3A). To validate the circRBMS3 aggregation ability regarding miRNAs in OS cells, we choose 11 candidate miRNAs by overlapping the anticipated findings of miRNA recognition elements in the circRBMS3 sequence by miRanda (Score Threshold > 140), Targetscan (at least 6mer binding site), and RNAhybrid (Figure 3B). After that, we looked at whether or not circRBMS3 was capable of directly binding these candidate miRNAs. The design and verification of a biotin-labeled circRBMS3 probe were performed in OS cell lines, and the pull-down efficiency was established (Figure 3C, 3D and Supplementary Figure 3B). The miRNAs extraction followed the pull-down assay, and the RT-qPCR determined the levels of 11 candidate miRNAs. As Figure 3E illustrates, the miR-15a/497/424 cluster was the only one abundantly pulled down by circRBMS3 in HOS and 143B cells. Because circRBMS3 contained miR-15a/497/424 binding sites (Figure 3F), we transfected miR-15a/497/424 mimics into HEK-293T cells and noticed the insertion of the circRBMS3 binding sequence in the 3’ untranslated regions (3’-UTR) downregulated luciferase activity (Figure 3G). Compared with controls, miR-424 lowered luciferase activity to the maximum degree (~45%) (Figure 3G). We then mutated the binding sites, and the 3 miRNAs transfection showed no significant impact on luciferase activity when the appropriate target locations in the luciferase reporter were mutated (Figure 3G). We then chose miR-424 for further investigation. FISH assays demonstrated the circRBMS3 interaction with miR-424 in OS cells cytoplasm (Figure 3H). These findings assumed the circRBMS3 sponge functions for miR-424.

### miR-424 suppresses OS cell migration, invasion, and proliferation *in vitro*

**Figure 4:**
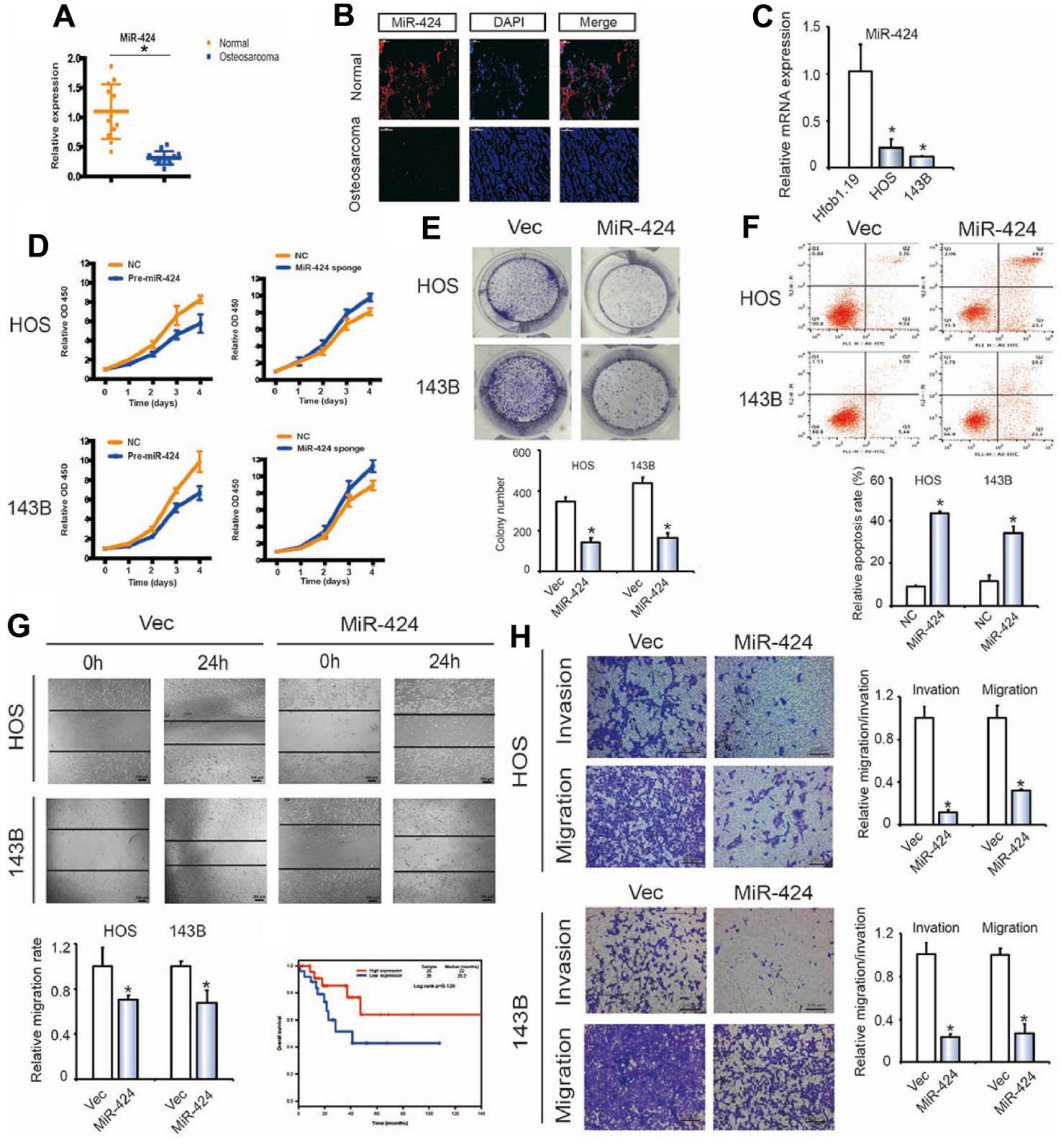
**miR-424 is associated with OS cell migration and invasion.** (**A**) MiR-424 expression was lower in human OS than in chondroma tissue. Data represent the mean ± SD (n = 12). (**B**) MiR-424 expression was lower in human OS than in chondroma tissue. Representative images are shown. (**C**) MiR-424 expression in hFOB1.19 and OS cell lines (143B and HOS) was evaluated by RT-qPCR. Data represent the mean ± SD (n = 3). * *P* < 0.05. (**D**) MiR-424 overexpression did not affect circRBMS3 and RBMS3 expressions. (**D**) Pre-miR-424 or miR-424 sponge mediated miR-424 overexpression and inhibition of OS cell proliferation, as determined by the CCK-8 assay. Data are presented as the mean ± SD (n = 6). (**E**) MiR-424 overexpression suppressed cell growth, as determined by the colony formation assay (details are shown in the insets). Error bars represent the mean ± SD of 3 independent experiments. * *P* < 0.05. (**F**) HOS and 143B cells were transfected with miR-424 mimics, followed by Annexin V-FITC/PI staining. The percentage of apoptotic cells is shown as the mean ± SD from 3 independent experiments. * *P* < 0.05, significantly different compared with the vector group. (**G**) The effect of pre-miR-424 on cell migration capability was evaluated by a wound-healing assay in HOS and 143B cells. Data are the mean ± SD, n = 3. * *P* < 0.05. Scale bar, 200 μm. (**H**) Cell migration and invasion of HOS and 143B cells, transfected with pre-miR-424 or vector, were evaluated by transwell migration and Matrigel™ invasion assays. Data represent mean ± SD (n = 3). * *P* < 0.05. Scale bar, 200 μm.

By performing qPCR and FISH, miR-424 downregulation was observed in human OS samples than in chondroma tissues (Figure 4A, 4B). MiR-424 was also reduced in OS cells than in hFOB1.19 cells (Figure 4C). We then investigated whether miR-424 has a tumor repressor role during OS cell proliferation. Pre-miR-424 or miR-424 sponges were transfected into HOS and 143B cell lines, as well as the control vector. The RT-qPCR verified miR-424 expression (Supplementary Figure 3C). CCK-8 and colony formation assay findings indicated the miR-424 overexpression in OS cells caused significant inhibition of cell proliferation (Figure 4D, 4E). Contrary, the proliferation rates of OS cells transfected with the miR-424 sponge showed more significant elevation than those transfected with the control vector (Figure 4D). The miR-424 ectopic expression caused a significant rise in the apoptosis rate 48 h after transfection of miR-424 mimics (Figure 4F). the wound healing assay (Figure 4G) and Transwell assay (Figure 4H) revealed the miR-424 upregulation reduced OS cells migration and invasion. The above findings confirmed that miR-424 decreased OS cells proliferation, migration, and invasion.

### *EIF4B* and *YRDC* are direct targets of miR-424

**Figure 5:**
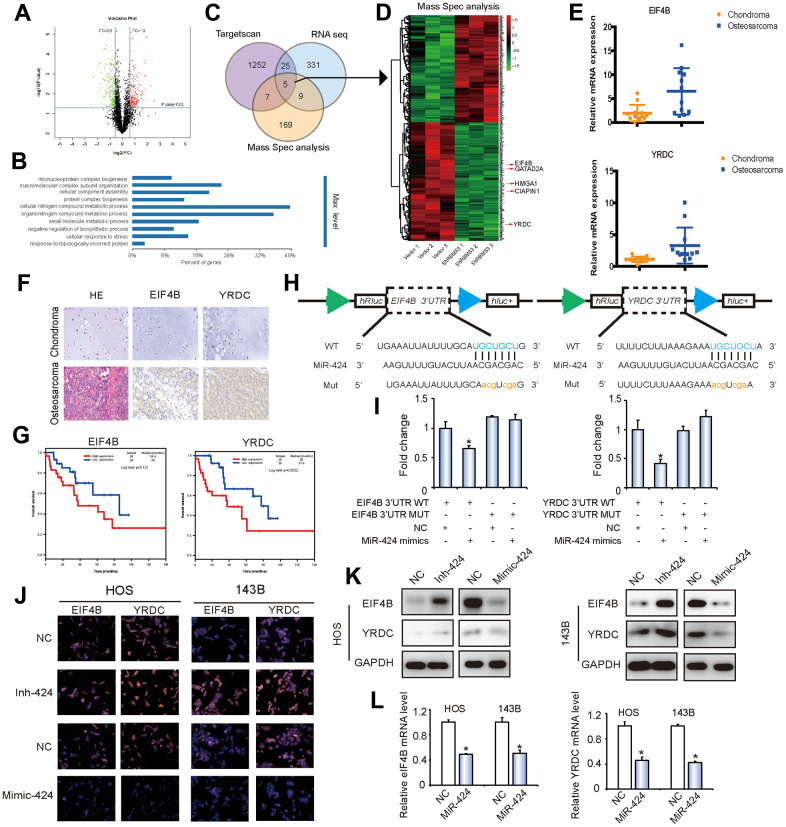
***EIF4B* and *YRDC* are direct targets of miR-424.** (**A**) Volcano plots of protein profiles. 143B Cells were transfected with shcircRBMS3, followed by protein profiles, with 3 repeats for each sample. (**B**) KEGG analysis of protein profiles. 143B cells were transfected with shcircRBMS3, followed by protein profiles, with 3 repeats for each sample. (**C**) Schematic illustration showing overlapping of the target mRNAs of circRBMS3 and miR-424 predicted by RNA-seq, Targetscan, and by Mass Spec analysis. (**D**) Heatmap of differentially expressed proteins after circRBMS3 knockdown. (**E**) *EIF4B* and *YRDC* expression levels were higher in human OS than in chondroma tissue examined by T test. Data represent the mean ± SD (n = 12). (**F**) EIF4B and YRDC expression levels were higher in human OS than in chondroma tissue. Representative images are shown. (**G**) Kaplan-Meier survival analysis of EIF4B and YRDC low and high sarcoma patients (log rank test). (**H**) Schematic illustration showing complementarity to the miR-424 seed sequence in the 3’-UTR of *EIF4B* and *YRDC*. Lowercase letters indicate mutated nucleotides. (**I**) 293T cells were co-transfected with pre-miR-424 and luciferase reporter constructs containing wild-type (WT) or mutated *EIF4B* and *YRDC* 3’-UTRs. Data represent the mean ± SD (n = 3). * *P* < 0.05. (**J**–**L**) MiR-424 overexpression reduced EIF4B and YRDC (**J**, **K**) protein and (**L**) mRNA levels while miR-424 inhibition increased EIF4B and YRDC (**J**, **K**) protein and (**L**) mRNA levels. Cells were transfected with NC or miR-424 mimic/inhibitor, and mRNA or protein levels evaluated. Protein expression was evaluated by western blot and immunofluorescence; mRNA levels were evaluated by RT-qPCR. Data represent the mean ± SD (n = 3). * *P* < 0.05. Scale bars = 50 μm.

Bioinformatics analysis was conducted to search for possible regulatory targets of miR-424. We got 1287 potential targets of miR-424 from Targetscan (https://www.targetscan.org) with the standard “at least 6mer binding sites”, 270 circRBMS3-regulating genes from RNA sequence and 190 circRBMS3-regulating proteins from Mass Spec analysis. We chose 5 candidate genes by overlapping the anticipated findings of gene recognition elements using mRNA sequencing, Targetscan, and Mass Spec analysis (Figure 5A–5D and Supplementary Figure 4A–4H). Among these genes, EIF4B and YRDC, whose inhibition leads to the highest apoptosis rates in OS, were selected as targets for further analysis (Supplementary Figures 4I, 4J). RT-qPCR and immunohistochemistry demonstrated the overexpression of EIF4B and YRDC in OS tissues compared to chondroma tissues (Figure 5E, 5F). Kaplan-Meier survival curves from the TCGA sarcoma dataset demonstrated that increased expression patients (cut-off by the median gene expression value) of EIF4B or YRDC had a lower 10-year overall survival rate (Figure 5G).

To verify whether *EIF4B* and *YRDC* are direct targets of miR-424, we designate 3’UTR sensors along with co-transfected HEK-293T cells with miR-424 mimic or NC (negative control). A reduction in luciferase activity of EIF4B and YRDC 3’ UTR was noticed in the miR-424 presence (Figures 5H, 5I). For verifying target specificity, we produced mutant variants of the *EIF4B* and *YRDC* 3’-UTR, where the miR-424-binding site was eliminated (Figure 5H). Co-transfection of miR-424 mimics alongside the mutant structure prevented the reduction in wild-type 3’-UTR luciferase activity, suggesting that miR-424 regulates EIF4B and YRDC expression in a particular manner (Figure 5I).

To determine if miR-424 could influence the eIF4B and YRDC expressions, HOS and 143B cells were transfected with miR-424 mimics, suppressors, or respective controls. The results of immunofluorescence (Figure 5J) and western blotting analyses (Figure 5K) showed that miR-424 mimics markedly suppressed eIF4B and YRDC protein levels in OS cells, while the miR-424 inhibitor clearly promoted eIF4B and YRDC protein expression. Moreover, following transfection, there was a similar downregulation in mRNA and protein levels of eIF4B and YRDC (Figure 5L). In summary, these results strongly suggest that miR-424 directly regulates eIF4B and YRDC in OS cell lines.

**Figure 6:**
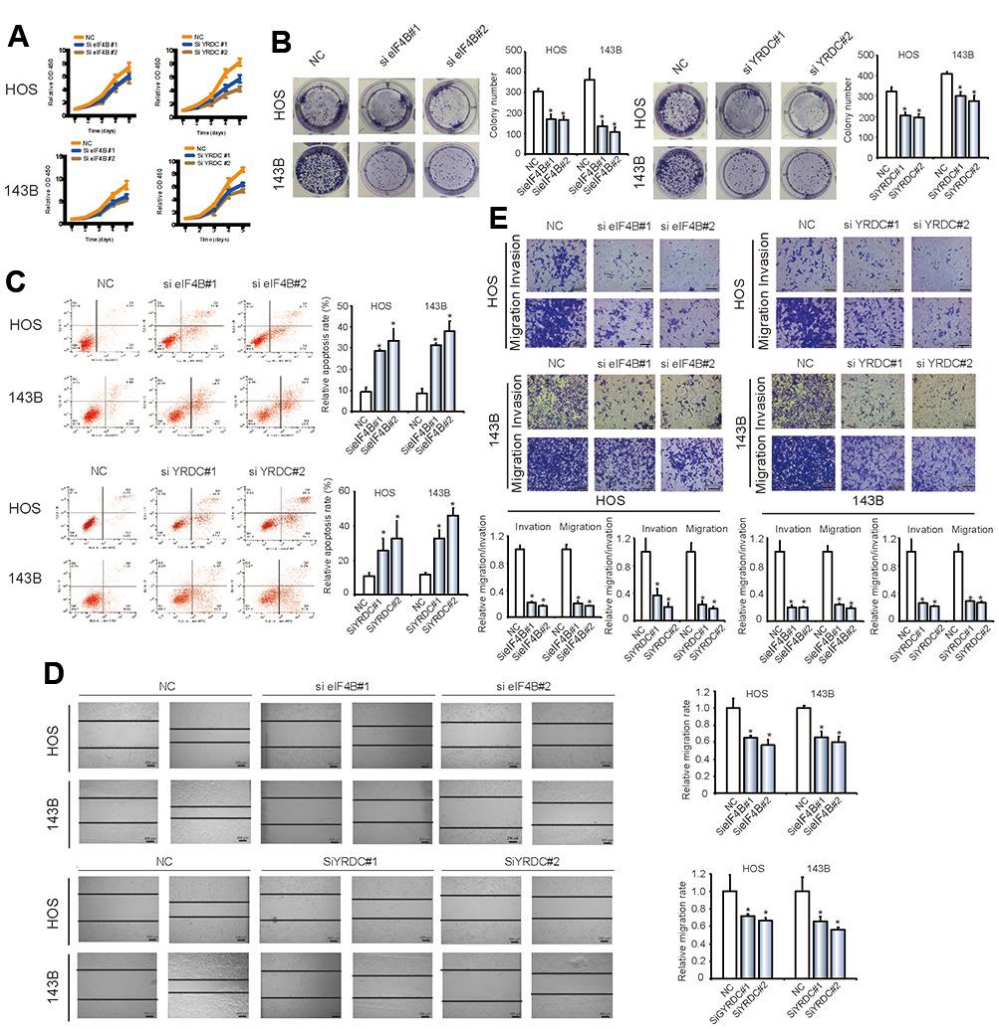
**EIF4B and YRDC inhibit the migration and invasion of OS cells *in vitro*.** (**A**) SiRNA-mediated *EIF4B* and *YRDC* knockdown suppressed OS cell proliferation, as determined in the CCK-8 assay. Data represent the mean ± SD (n = 6). (**B**) *EIF4B* and *YRDC* knockdown suppressed cell growth, as determined by the colony formation assay (details are shown in the insets). Error bars represent the mean ± SD of 3 independent experiments. * *P* < 0.05. (**C**) HOS and 143B cells were transfected with *EIF4B* or *YRDC* siRNA, followed by Annexin V-FITC/PI staining. The percentage of apoptotic cells is shown as the mean ± SD from the 3 independent experiments. * *P* < 0.05, significantly different compared with the vector group. (**D**) The effect of *EIF4B* or *YRDC* siRNA on cell migration capability was evaluated by a wound-healing assay using HOS and 143B cells. Data are mean ± SD, n = 3. * *P* < 0.05. Scale bar, 200 μm. (**E**) EIF4B or YRDC knockdown suppresses cell migration and invasion abilities of HOS and 143B cells, as evaluated by Transwell migration and Matrigel invasion assays. Data represent the mean ± SD (n = 3). * *P* < 0.05. Scale bar, 200 μm.

EIF4B and YRDC are known as oncogenes in some tumor types, while their function in OS is currently not understood. We explored the effect of eIF4B and YRDC on OS tumorigenesis by CCK-8 and colony formation experiments. There was a significant reduction in proliferation after inhibition of eIF4B and YRDC expression (*P < 0.05; Figure 6A, 6B). Furthermore, the knockdown of eIF4B and YRDC clearly induced higher levels of apoptosis (Figure 6C, *P < 0.05). In addition, the wound healing (Figure 6D) and transwell assays (Figure 6E) revealed the inhibition of eIF4B and YRDC reduced tumor cells migration and invasion. The above findings suggest that eIF4B and YRDC are tumorigenic and are direct targets of miR-424.

### Knockdown of miR-424 reverses shcircRBMS3-induced attenuation of OS cell proliferation, migration, and invasion

**Figure 7:**
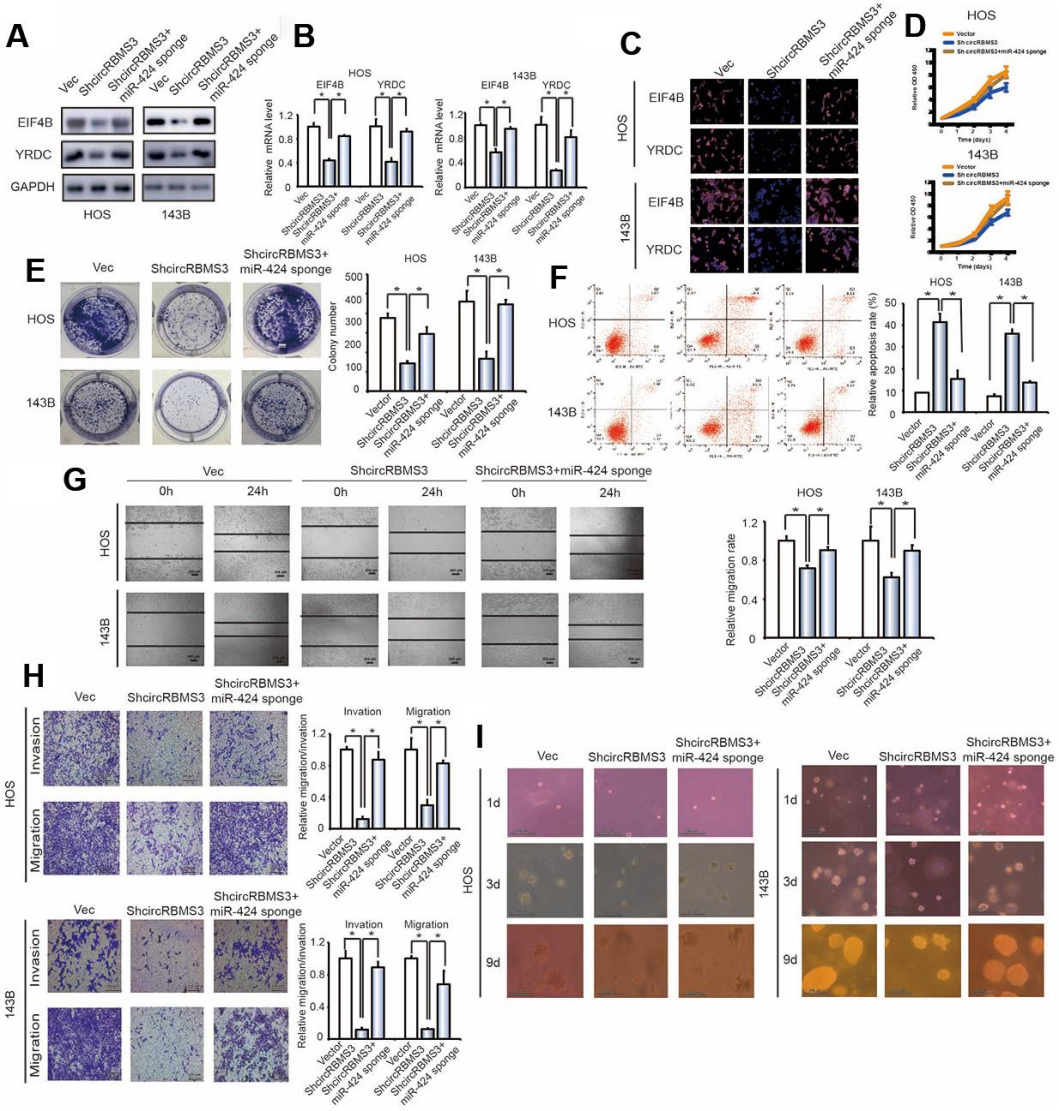
**Knockdown of miR-424 reverses shcircRBMS3-induced attenuation of cell proliferation, migration, and invasion in OS cells.** (**A**) The expression of EIF4B and YRDC in HOS and 143B cells was detected by western blot analysis. Cells were co-transfected with shcircRBMS3 and miR-424 sponge or control vector. Data represent the mean ± SD (n = 3). (**B**) The mRNA expression of *EIF4B* and *YRDC* in HOS and 143B cells was detected by RT-qPCR analysis. Cells were transfected with control vector and shcircRBMS3 with or without miR-424 sponge. Data represent the mean ± SD (n = 3). * *P* < 0.05 (**C**) The expression of EIF4B and YRDC in HOS and 143B cells was detected by immunofluorescence analysis. Cells were transfected with control vector and shcircRBMS3, with or without miR-424 sponge. Data represent the mean ± SD (n = 3). Scale bars = 50 μm. (**D**) Proliferation of OS cells transfected with control vector and shcircRBMS3, with or without miR-424 sponge, was evaluated by the CCK-8 assay. Data represent the mean ± SD of three independent experiments. (**E**) miR-424 downregulation rescued the growth inhibition of circRBMS3 knockdown in OS cells, as determined by colony formation assays (details are shown in the insets). Data represent the mean ± SD (n = 3). * *P* < 0.05. (**F**) Downregulation of both circRBMS3 and miR-424 resulted in fewer apoptotic cells in OS cells, compared with circRBMS3 inhibition alone. Apoptosis rates were determined by Annexin V-FITC/PI staining and FACS. Data represent the mean ± SD (n = 3). * *P* < 0.05. (**G**) The downregulation of circRBMS3 and miR-424 on cell migration capability was evaluated by a wound-healing assay in HOS and 143B cells. Data represent mean ± SD (n = 3). * *P* < 0.05. Scale bar, 200 μm. (**H**) Effects of circRBMS3 inhibition on cell migration and invasion were eliminated by miR-424 downregulation. Migration and invasion of OS cells transfected with control vector and shcircRBMS3, with or without miR-424 sponge, were evaluated by the Matrigel™ and transwell invasion assays. Scale bars = 50 μm. (**I**) OS cells transfected with control vector and shcircRBMS3 with or without miR-424 sponge were cultured in soft agar for 20 days. Colonies were photographed.

We co-transfected the miR-424 sponge and the circRBMS3 knockdown constructs into OS cells. There was a significant overexpression in protein and mRNA of eIF4B and YRDC in OS cells co-transfected with shcircRBMS3 plasmid and the miR-424 sponge than in cells transfected with shcircRBMS3 alone (Figure 7A, 7B). Immunofluorescence investigation verified the overexpression of eIF4B and YRDC in 143B and HOS cells transfected with shcircRBMS3 and a miR-424 sponge contrasted to shcircRBMS3 alone (Figure 7C). The miR-424 and circRBMS3 knockdown caused a more elevated growth rate than the circRBMS3 suppression group (Figure 7D). The miR-424 and circRBMS3 downregulation enhanced colony formation more than cells transfected with shcircRBMS3 alone (*P < 0.05 for both; Figure 7E). Furthermore, apoptotic cell numbers were decreased after treatment with miR-424 sponge and shcircRBMS3 in co-transfected cells compared with shcircRBMS3 transfected cells (Figure 7F). Furthermore, wound healing assays (Figure 7G) and transwell Matrigel™ invasion experiments (Figure 7H) revealed the enhancement to invasion and migration capabilities caused by OS cells co-transfected with the miR-424 sponge and shcircRBMS3 expression constructs when compared to shcircRBMS3-transfected cells. Strikingly, we observed that inhibition of miR-424 in OS cells significantly strengthened the anchorage-independent growth ability of circRBMS3 knockdown cells (Figure 7I). The findings mentioned above suggested that circRBMS3 increases cell migration, invasion, and proliferation through sponging miR-424 and then stimulates the expression of EIF4B and YRDC *in vitro*.

### circRBMS3 functions as a miR-424 sponge to promote tumorigenesis *in vivo*

**Figure 8:**
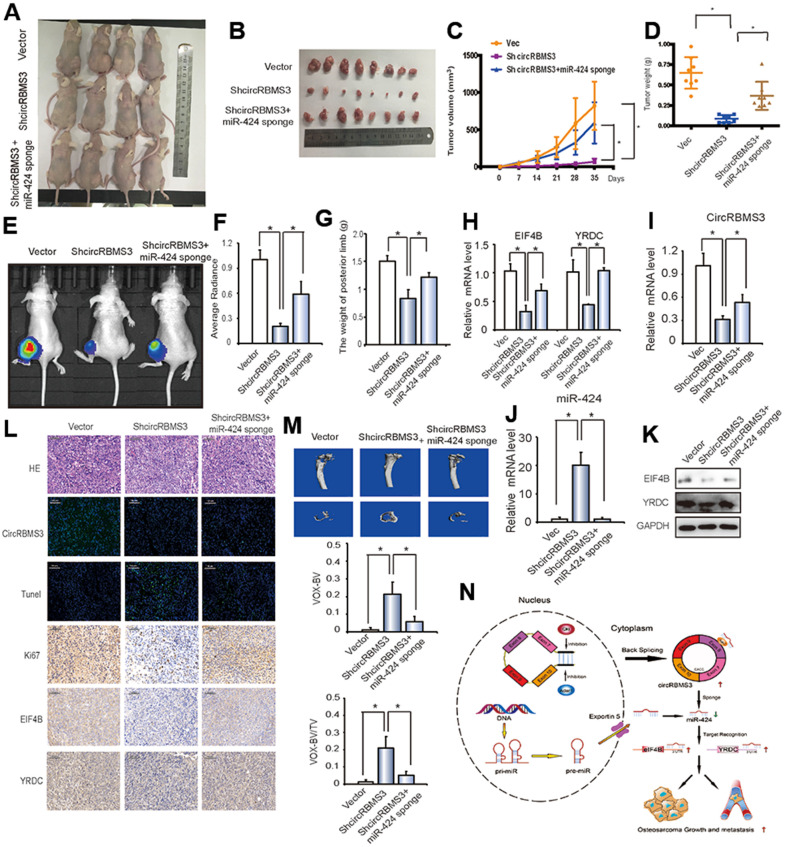
**circRBMS3 functions as a miR-424 sponge to promote tumorigenesis *in vivo*.** (**A**, **B**) Nude mice were injected with 5 × 10^6^ 143B stable cells. Four weeks later, tumors were dissected and photographed. (**C**) The graph represents tumor volumes (v = ab^2^/2) at injection with control cells or cells transfected with circRBMS3 short hairpin (sh)-RNA or co-transfected with circRBMS3 shRNA and miR-424 sponge (n = 6 per group). Data represent the mean ± SD (n = 8). (**D**) Average tumor weight in each group at the end of the experiment. Data represent the mean ± SD (n = 8). * *P* < 0.05. (**E**) *In vivo* imaging of tibia tumor. (**F**) Average radiance of orthotopic xenograft nude mice. (**G**) The limb weight of orthotopic xenograft nude mice. (**H**) RT-qPCR analysis of *EIF4B* and *YRDC* expression in tumors from xenograft mice. (**I**, **J**) Knockdown efficiency of circRBMS3 and miR-424 in tumors from orthotopic xenograft nude mice. (**K**) Western blot analysis of EIF4B and YRDC in tumors from xenograft mice. (**L**) Histological analysis of tumor tissues by hematoxylin and eosin staining. EIF4B and YRDC expression was examined by immunohistochemistry. Representative images are shown. (**M**) MicroCT quantification of the specific trabecular bone volume [BV/TV (%)] and the cortical BV (mm^3^)] were calculated for the tibia of tumor-bearing mice in the different groups. (**N**) Schematic illustration of the circRBMS3/miR-424 axis.

To explore the circRBMS3 and miR-424 effects *in vivo*, nude mice followed injection subcutaneously for 35 days with 143B cells transfected with circRBMS3-deficient, miR-424 sponge, or vector, and then tumor volumes were measured. Cells lacking miR-424 and circRBMS3 showed a higher tumor growth rate when compared to circRBMS3-deficient stable cells (Figure 8A, 8B). Similarly, co-expression of miR-424 inhibition and circRBMS3 knockdown constructs rescued the volume of 143B-derived tumors *in vivo* (Figure 8C), compared with the circRBMS3-deficient group alone. We observed the same findings for mean tumor wet weight across all 3 groups (Figure 8D).

For further evaluation of the circRBMS3 antitumor influence *in vivo*, we designated an orthotopic OS model by injecting 143B cells intra-tibial. The mice followed intra-tibial injection with circRBMS3-deficient, miR-424 sponge, or stable vector cells. As indicated in Figures 8E, 8F, *in vivo* imaging data demonstrated the growth inhibition of *in situ* tumors by the circRBMS3, while mice with tumors lacking both miR-424 and circRBMS3 had a higher tumor size and posterior limb weight (Figure 8G).

The circRBMS3 and two target genes relationship was then assessed *in vivo*. CircRBMS3 knockdown caused downregulation of the *eIF4B* and *YRDC* mRNA levels (Figure 8H). This corresponded to a reduction of eIF4B and YRDC expression, as determined by western blot (Figure 8K). Moreover, inhibition of both miR-424 and circRBMS3 rescued these effects (Figure 8H–8K). As shown in Figure 8L, circRBMS3 knockdown in tumor tissues resulted in a significant overexpression of terminal dUTP nick end labeling (TUNEL)-positive cells, whereas the levels of Ki67, eIF4B or YRDC were decreased, while inhibition of miR-424 reversed these effects.

Furthermore, the circRBMS3 impact on the bone microarchitecture of the tumor-bearing tibia was investigated by micro-computed tomography (CT) *in vivo*. As a result, circRBMS3 knockdown significantly reduced bone destruction (Figure 8M). The trabecular bone volume (BV/TV; from 0.013 to 0.208) was significantly improved after circRBMS3 knockdown (Figure 8M). Similar tendency was noticed for the BV (12.3 mm^3^ in the control group compared to 21.3 mm^3^ in the treated group), while miR-424 inhibition rescued these effects (Figure 8M). The abovementioned findings suggested that the circRBMS3 sponge role for miR-424 and that miR-424 governs the circRBMS3 tumorigenic function *in vivo* (Figure 8N).

## DISCUSSION

circRNAs are a new family of persistent and widespread endogenous RNAs governing mammalian gene expression [34]. Compared to linear RNA, circRNAs have more stability and resistance to RNA exonuclease or RNase due to their covalently-closed loop structures [35]. CircRNAs may be crucial in several malignancies, such as gastric and lung cancer, hepatocellular and colon carcinoma, sarcoma, and leukemia [36]. Due to the cell type and the specific features of the developmental stage, circRNAs may be vital in OS progression, offering novel therapeutic targets for OS treatment.

The differentially expressed circRNAs between OS and chondroma tissues were screened by RNA-seq, focusing on the function as well as the mechanism behind circRBMS3 overexpression in OS progression. RBMS3 (known as RNA binding motif), an interacting protein 3 with a single strand, belongs to the MSSP protein family. RBMS3 is widely expressed from embryonic stages through to adulthood and can promote fibrosis of the liver and participate in the formation of cartilage. RBMS3 is considered a novel malignancies inhibitor gene, including in breast cancer, esophageal squamous cell carcinoma, and nasopharyngeal carcinoma [23–26], thus attracting widespread attention. In particular, the level of RBMS3 in OS has not been investigated. Our research found a significant overexpression in the circRBMS3 levels in OS, while a downregulation in mRNA together with protein levels of RBMS3. In addition, *in vitro*, functional assays demonstrated the knockdown of circRBMS3 could enhance OS cell apoptosis and inhibit OS cell proliferation, invasion, and migration. Based on past research, we confirmed that ADAR1 could downregulate the formation of circRBMS3 but have no effects on the mRNA.

We demonstrated the upstream signal of circRBMS3 and further investigated the downstream effectors of circRBMS3. In comparison to circRNAs, miRNAs are hugely well-studied. Dysregulation of miRNA has been identified to have a close association with tumorigenesis. Generally, miRNAs decrease mRNA translation when they bind to the 3’-UTR of target genes [37], and they are also crucial in OS progression and metastasis [38]. Notably, circRNAs mainly function as miRNA sponges, leading to miRNA function loss and elevated their endogenous targets levels [39]. CeRNA networks are complicated, and the interactions between circRNAs and miRNAs have already been implicated as playing essential roles in a variety of cancers. An important example is CDR1 as a unique circRNA, for which exceeding 70 binding sites of miR-7 have been identified. CDR1as thus has an enormous capability to suppress miR-7 activity [19]. Another circRNA, termed circHIPK3, derived from exon 2 of HIPK3, serves as miR-558 and miR-124 sponge [40]. We screened multiple anticipated binding miRNAs via bioinformatics analysis, confirming the miR-424 ability to bind with circRBMS3 via a biotin-coupled probe pull down assay. MiR-424 is reported to be a tumor inhibitor in multiple cancers [41–43]. MiR-424 suppresses the progression of OS by directly targeting YRDC and eIF4B in OS, as demonstrated by our functional investigations and luciferase reporter assay.

Consequently, we reveal a novel regulatory axis created byADAR1-circRBMS3-miR-424-YRDC/eIF4B in OS. Taken together, we are able to confirm the circRBMS3 regulatory function and its sponging influence on miRNAs in OS depending on functional and molecular analyses. These findings are in full accordance with our hypothesis that circRBMS3 protects YRDC/eIF4B and promotes OS progression by sponging tumor-suppressive miRNAs.

RNA binding protein is a tumor suppressor gene inhibiting various tumor progression, including glioblastoma, oral squamous cell carcinoma, gastric cancer, astrocytic glioma, and colon cancer [44, 45]. In eukaryotes, RNA editing is an essential post-transcriptional RNA mechanism generating RNA and protein diversity. A typical form of RNA editing is adenosine-to-inosine editing, which is dependent on RNA-acting members of the adenosine deaminase family (ADARs). In mammals, ADAR1 exists in many tissues, can edit non-coding RNAs, influences their biogenesis, and modify their target gene specificity. ADAR1 affects various biological processes, including miRNA processing [46], forming protein–protein complexes [47], and influencing gene expression [48]. ADAR1 has also been shown to be downregulated in melanoma development, and ADAR1-editing miRNA-455-5p can inhibit melanoma growth and metastasis *in vivo* [49]. However, the ADAR1 function in malignancies is still unobvious. The ADAR1 expression and role in the human OS have never been mentioned before. We discovered the significant downregulation of ADAR1 in mRNA and protein levels in OS cells, besides the ADAR1 inhibition ability on the circRBMS3 expression levels, assumed its tumor-suppressive function in OS. Nevertheless, ADAR1 obviously edits other circRNAs or lncRNAS in OS progression. Therefore, the function of ADAR1 requires further exploration. Nuclear RNA helicase DHX9 is widely distributed and mostly attaches to inverted-repeat Alu elements, suppressing circRNAs production when it binds to their flanking inverted complementary sequences and inhibits their pairing [31]. DHX9 is essential in OS development [31–33]; however, in the current study, DHX9 knockdown did not affect the expression of circRBMS3 or RBMS3 mRNA.

Eukaryotic translation initiation factor 4B (eIF4B), which is vital in ribosomal scanning via structured mRNA leaders [50, 51], has been shown its involvement in translating several proliferative or anti-apoptotic mRNAs with highly structured 5’ UTRs and thereby influences cell growth and survival [52]. Abnormal eIF4B levels or phosphorylation are closely related to various tumors, including breast cancer, leukemia, Kaposi’s sarcoma, and lymphoma [53–55]. YRDC is ubiquitously expressed in human tissues, especially in the pancreas and liver. Recent studies have indicated that YRDC functions as an oncogene and can promote cell proliferation in bladder cancer [56]. Nevertheless, we do not know much about the biology of these two genes in OS. To study the function of YRDC and eIF4B in OS, YRDC or eIF4B were knocked down using specific siRNAs. We found that either si-YRDC or si-eIF4B could lead to suppressive effects on OS cell proliferation and migration. Furthermore, a luciferase reporter assay, immunofluorescence (IF), western blotting (WB), or RT-qPCR experiments were employed to validate further that YRDC and EIF4B are direct miR-424 targets.

In conclusion, the current study investigates the regulatory function of a newly-found circRBMS3 (hsa_circ_0064644) that is overexpressed in human OS as well as can efficacy sponge miR-424 to enhance YRDC/eIF4B expression. Through the circRBMS3- miR-424-YRDC/eIF4B axis, circRBMS3 performed specific regulatory roles affecting the tumorigenesis of OS. In this study, although miR-424 significantly inhibited OS cell migration and invasion, the miR-424 sponge could only partially reverse the effects induced by circRBMS3 knockdown in *in vivo* study. However, CircRBMS3-miR-424-YRDC/eIF4B axis is actually essential in OS proliferation and invasion. Hence, it is reasonable to assume that other genes or regulatory pathways may participate in the roles of circRBMS3 in OS tumorigenesis. CircRBMS3 might serve as a new biomarker regarding poor prognosis in OS. Furthermore, the circRBMS3- miR-424-YRDC/eIF4B axis is a new signaling pathway with the potential treat OS patients. Our study also provides novel evidence to suggest that circRNAs act as “microRNA sponges”, offering a new OS treatment.

## CONCLUSIONS

Our results reveal an essential role for a novel circRBMS3 in OS cell growth and metastasis, providing a fresh point of view on circRNAs in the progression of OS. Mechanistically, our *in vitro* as well as *in vivo* experiments revealed the CircRBMS3 role as a ceRNA for miR-424 and governed the EIF4B and YRDC expression in OS progression. Thus, the critical role of circRBMS3-miR-424-eIF4B/YRDC axis may appear to be a new diagnostic and therapeutic target for OS.

## MATERIALS AND METHODS

### Cell culture and transfection

DMEM-F12 medium (Gibco) was used for the Human fetal osteoblasts hFOB1.19 culture. RPMI 1640 medium (Gibco) with 10% fetal bovine serum (FBS) (Gibco) and antibiotics (100 IU/mL penicillin as well as 100 lg/mL streptomycin) was utilized for the Human OS cell lines HOS and 143B culture that followed standard techniques in an incubator with humidity and 5% (v/v) CO2.

### Analysis of circRNA expression profile

Between 2016 and 2018, at the Department of Orthopaedic Surgery of The Second Affiliated Hospital of Zhejiang University (Hangzhou, China) and Sir Run Run Shaw Hospital, Medical College of Zhejiang University (Hangzhou, China) with permission from the Ethics Committee, respectively, our group obtained 12 osteoblastic OS (IIB stage) and 12 chondroma tissues from patients and then analyzed them via the circRNAs chips. Kang Chen Biotech, Shanghai, China, hybridized the microarray and collected data. On the bias of circRNAs expressing values, we performed the top five overexpressed and downregulated circRNAs with their hierarchical clustering assessment via the Cluster and TreeView program.

### Cell culture

The purchase of hFOB1.19, human OS, HOS, and 143B cell lines were from FuHeng Cell Center (Shanghai, China). ShangHai Biowing Applied Biotechnology Co. Ltd authenticated the OS cell lines, based on STR profiling analysis in accordance with the ATCC Standards Organization's Capes-Davis description and the ANSI Standard (ASN-0002). The Venor GeM Mycoplasma Detection Kit (Minerva Biolabs, Berlin, Germany) was used for the mycoplasma testing. Detailed tissue culture methods, as well as details of transfection and viral infection, can be found in the Supplementary Information.

### RNA extraction and quantitative real-time polymerase chain reaction (qRT-PCR)

TRIzol Reagent (Invitrogen) performed the total RNA extraction from OS tissues and cells. The divergent primer performed CircRNAs amplification, and the RNase R was utilized for linear RNA degradation. The prime Script RT reagent Kit (TaKaRa) together with SYBR Premix Ex Taq II (TaKaRa) carried out the QRT-PCR analysis on both CircRNAs and mRNA. As a control, β-actin was used. Regarding miR-424 assessment, the treatment of miRNA with DNase I was performed for genomic DNA elimination, while Mir-X miR First-Strand Synthesis Kit (TaKaRa) performed cDNA synthesis with U6 as a standard internal control. A thrice replication was done to every sample, and the Ct values comparison performed the data analysis.

### Cell proliferation and cloning formation assays

For the cell proliferation assay, 96-well plates were seeded with 2,000 transfected cells/well. At 0, 24, 48, 72, and 96 h following seeding, the cell counting kit-8 (CCK-8) system (Dojindo, Japan) performed the cell viability measurement per the protocols. The 10 μl CCK-8 solution addition into every well was followed by a 37° C incubation of the plate for one hour in darkness. Moreover, a microplate reader (Tecan, Switzerland) measured the absorbance at 450 nm for every well.

For the colony formation assay, the seeding of 400 transfected OS cells took place onto 6-well plates. Following 2-week incubation, the cells underwent fixation and staining with methanol as well as 0.1% crystal violet, respectively. Then we got images for the colonies and counted them.

### Ago2-binding sites from CLIP data sets

The online cross-linking immunoprecipitation (CLIP) data sets comprising Ago2 HITS-CLIP as well as PAR-CLIP data from several lymphoma cells along with HEK-293 cells. The download of the available data sets was from doRiNA (an RNA interactions database in post-transcriptional regulation, http://dorina.mdc-berlin.de/regulators), and the Ago2-binding sites of circRBMS3 genomic region were acquired.

### Subcutaneous and orthotopic xenograft OS mouse models

Nude mice (nu/nu, male aged 3-4 weeks) followed subcutaneous or tibial medullary cavity injection of 5 × 10^6^ 143B stable cells. This formula: volume (ml^3^) = ab^2^/2, was used for tumor volumes calculation where (a) is for length and (b) for width. After a Five-weeks injection followed by mice euthanize, tumors were harvested, measured, weighed, and then underwent 4% paraformaldehyde fixation. Each group's wet tumor weight was calculated as the mean weight ± standard deviation (SD).

### MicroCT analysis

A high-resolution microcomputed tomography (Skyscan 1076) was used for scanning the tibias at 50 kV and 200 mA with a 0.5 mm aluminum filter. Moreover, every 0.6° during 180° of rotation, images were captured, and Skyscan software performed their analysis. Quantification of trabecular structures was performed across a length of one mm beginning 0.2 mm below the growth plate. Bone volume (BV)/tumor volume (TV) and bone volume (BV) of cortical bones were assessed.

### RNA immunoprecipitation

Utilizing the magna RIP RNA-Binding Protein Immunoprecipitation Kit (Millipore, Bedford, MA, USA), we carried out the Ago-RIP assay in HOS cells. The circRBMS3 siRNAs and sicontrol transfection into HOS was done first. The sedimentation and resuspension of about 1 × 10^7^ cells were with an equivalent RIP Lysis Buffer pellet volume with a protease blocker cocktail as well as RNase blockers. After incubating cell lysates (200 μl) with 5 μg of anti-Ago2 antibody (Millipore, Billerica, MA, USA) or control rabbit IgG-coated beads, the mixture was then rotated at 4° C overnight. Following treatment with proteinase K buffer, RNeasy MinElute Cleanup Kit (Qiagen, Duesseldorf, Germany) performed the immunoprecipitated RNAs extraction, while the reverse transcription was by Prime- Script RT Master Mix (TaKaRa, Tokyo, Japan). The RT–qPCR assay detected the circRBMS3 abundance.

### Luciferase reporter assay

The seeding of HEK293T cells took place into 96-well plates and underwent culture to 50–70% confluence prior to transfection. For circRBMS3 and miR-424, 600 ng plasmids of circRBMS3-wt and circRBMS3-mut, 20 nmol miR-424 and N.C. were transfected. Following incubation for 48 hours, the detection of firefly and Renilla luciferase activities was conducted by the Promega Dual-Luciferase system. For providing an internal reference, firefly luciferase activities were evaluated with a 100 ml Luciferase Assay Reagent II (LAR II) (Luciferase Assay Reagent, Promega), and then 20 ml lysis buffer, moreover 100 ml Stop and Glo® Reagent (Luciferase Assay Reagent, Promega) was utilized for evaluating Renilla luciferase activities. Calculation of the difference between a firefly and Renilla luciferase activities was to evaluate relative luciferase activity.

### Cell apoptosis assays

For detecting cell apoptosis, an annexin V-FITC/PI apoptosis kit (BD Biosciences, San Jose, CA, USA) performed the staining, and flow cytometry analyzed them. In each experiment, a comparison between early and late apoptotic cell ratio and the obtained values from the controls was performed.

### Western blot

Utilizing the radioimmunoprecipitation assay buffer (RIPA, Beyotime, China) together with bicinchoninic acid (BCA) analysis (Beyotime, China), the cell lysis and protein harvesting, and quantification were performed, respectively. The separation of extracted proteins was by 10% SDS-PAGE, and the transfer was to polyvinylidene fluoride (PVDF) membranes (Sigma-Aldrich, USA). Following being incubated with high-affinity antibodies, including anti-eIF4B (1:1000) (abcam), anti-YRDC (1:1000) (abcam), and anti-GAPDH (1:2000) (Cell Signaling Technology, USA), other membranes incubation with a secondary antibody (1:5000, Cell Signaling Technology, USA) took place. Upon washing, the signal detections were performed by a chemiluminescence system (Bio-Rad, USA), followed by their analysis via Image Lab Software.

### Northern blot

The Northern blot analysis was conducted using Northern blot kit (Ambion, USA). The denature of almost total RNAs (30 μg) of OS cells was within formaldehyde and then underwent 1% agarose–formaldehyde gel electrophoresis. Then followed by an RNAs transfer to a Hybond-N + nylonmembrane (Beyotime, China) and hybridization using biotin-labeled DNA probes. A biotin chromogenic detection kit (Thermo Scientific, USA) helped in the detection of bound RNAs. The membranes underwent exposure and analysis via Image Lab software (Bio-Rad, USA).

### RNA *in situ* hybridization

RNA in situ hybridization: MiR-424 probe labeled by Cy3 and CircRBMS3 probe labeled by Alexa Fluor 488 were produced by RiboBio technology (Guangzhou, China). RNA FISH staining was performed according to manufacturer’s instructions. The specimen analysis was performed on a Nikon inverted fluorescence microscope.

### Pull-down assay with biotinylated circRBMS3 probe

The 1 × 10^7^ HOS and 143B cell harvest followed by lysing and sonicate. For generating probe-coated beads, a 2 h incubation of circRBMS3 probe with C-1 magnetic beads (Life Technologies, Gaithersburg, MD, USA) at 25° C was performed. Incubating cell lysates overnight at 4° C with circRBMS3 or oligo probes. Following the washing buffer, RNA complexes were eluted and extracted for RT–qPCR or qPCR experiments. RiboBio (Guangzhou, China) was used in constructing and synthesizing the Biotinylated-circRBMS3 probe.

### Wound healing assay

Six-well plates were used for HOS and 143B cells culture, and the tip of a 200 μl pipette was used for scraping at time point 0. Cells migrating into ‘wounded’ areas were assessed at 24 h by an inverted microscope (Olympus, Tokyo, Japan) along with microphotographed. The healing rate quantification was determined by the extent of gap sizes.

### Transwell migration and Matrigel™ invasion assays

200 μl of the serum-free medium was used as a suspension for approximately 5 × 10^4^ of the transfected cells, and then the upper chambers of every transwell (8 μm pore size, Costar, NY, USA) were used for seeding them; those chambers were covered with or without Matrigel (BD Biosciences, San Jose, CA, USA) for the two assays. The bottom chamber included 10% FBS medium as a chemoattractant. Following 37° C incubation with 5% CO_2_ for 48 and 24 h of the invasion and migration assay, respectively, Cotton swabs were used to remove cells in the upper chamber, while for the cells in the lower surface, fixation and 0.1% crystal violet staining were performed. Migration and invasion rates were calculated by cell counts in minimum 3 random fields.

### Statistical analysis

SPSS v22.0 software carried out the statistical analyses. The independent sample t-tests performed the two groups comparison, while the one-way analysis of variance (ANOVA) utilized an LSD-t for multiple group comparisons (equal variance assumed). *P* < 0.05 was regarded as statistically significant.

### Consent for publication

Obtained.

### Availability of supporting data

The datasets used and/or analyzed during the current study are available from the corresponding author on reasonable request.

## Supplementary Material

Supplementary Figures

Supplementary Table 1
