# Supplementary material for: A novel circular RNA circRBMS3 regulates proliferation and metastasis of osteosarcoma by targeting miR-424-eIF4B/YRDC axis
Source: Aging (Albany NY). 2023 Mar 9;15(5):1564–90. doi: 10.18632/aging.204567 (PMC10042691; doi:10.18632/aging.204567)
Supplement: Supplementary Figures [file aging-15-204567-s001.pdf]

SUPPLEMENTARY FIGURES

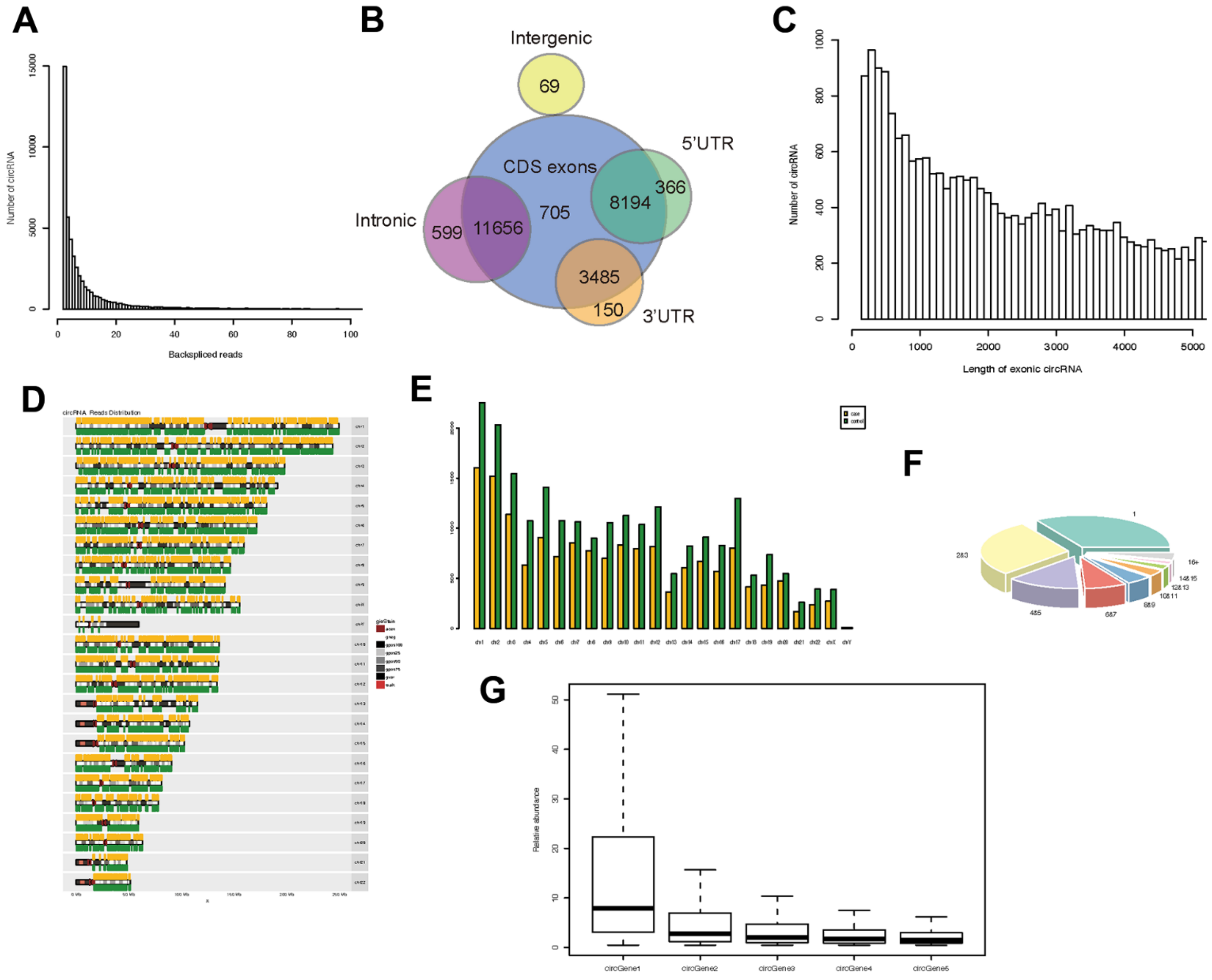

Supplementary Figure 1. Profiling of circular RNAs in human OS and chondroma tissues. (**A**) RNA-seq read abundance distribution of identified circular RNAs (circRNAs); x-axis, the back-spliced read numbers of circRNAs detected by RNA-seq; y-axis, the abundance of circRNAs classified by different read numbers. The majority of named circRNAs in the study were supported by more than 10 reads. (**B**) Venn plot showing the number of circRNAs derived from different genomic regions. (**C**) Length distribution of the identified circRNAs; x-axis, the length of circRNAs detected in this study; y-axis, the abundance of circRNAs classified by length. (**D**) The distribution of identified circRNAs in chromosomes. The yellow and cyan bars represent the location of detected circRNAs within different chromosomes in chondroma and tumor samples, respectively. (**E**) Number of circRNAs produced from one gene. (**F**) Numbers of identified circRNAs in different chromosomes. The yellow and cyan bars represent the numbers of circRNAs within different chromosomes detected in chondroma and tumor samples, respectively. (**G**) Predominant circRNAs analysis of all samples.

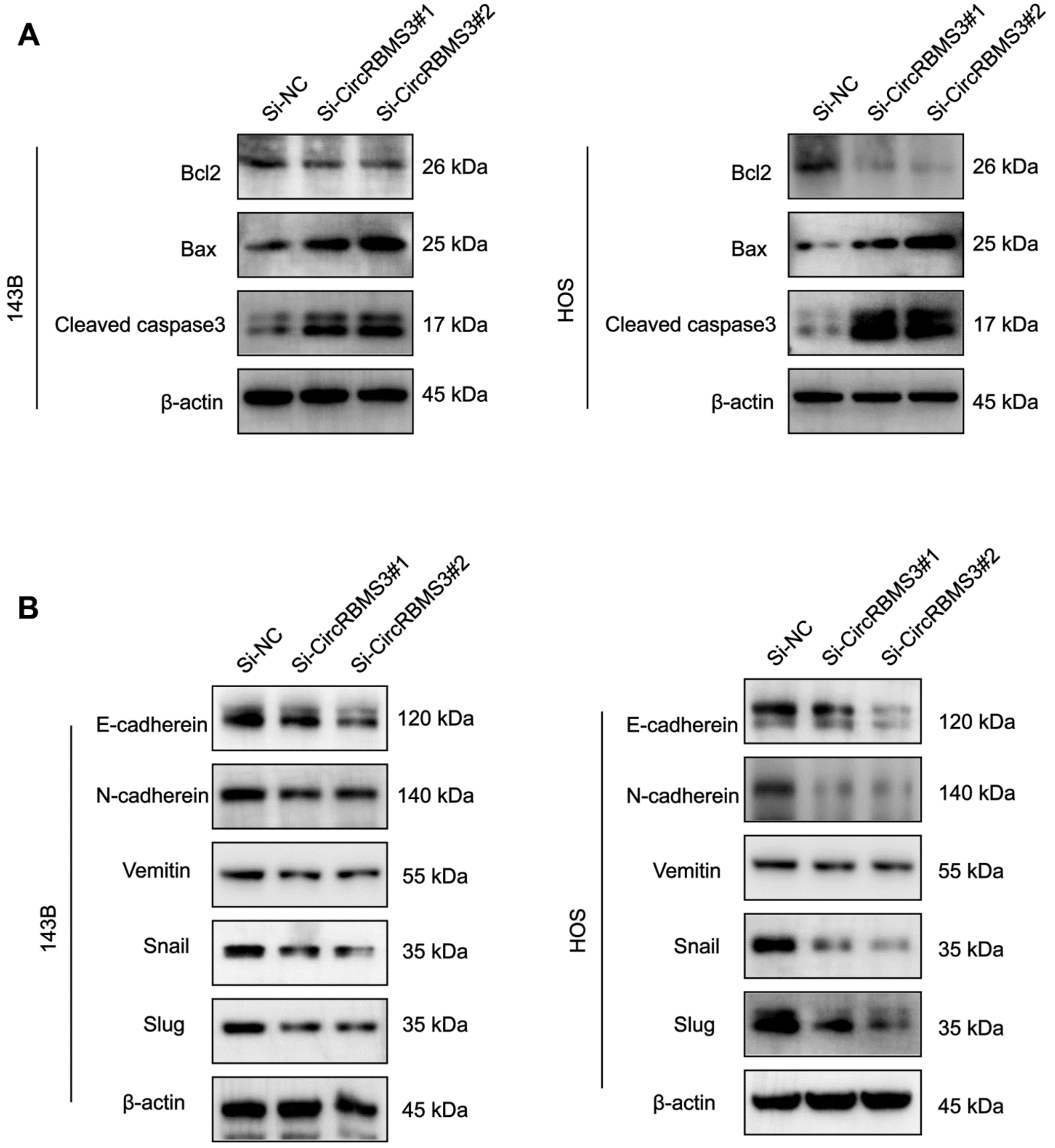

Supplementary Figure 2. circRBMS3 knockdown promotes OS cells apoptosis and reduces OS cells invasion. (**A**) Bcl2, Bax, and cleaved caspase3 proteins expression in 143B and HOS cells after circRBMS3 knockdown indicated by Western blot. (**B**) E-cadherein, N-cadherein, Vemitin, Snail, and Slug proteins expression in 143B and HOS cells after circRBMS3 knockdown indicated by Western blot.

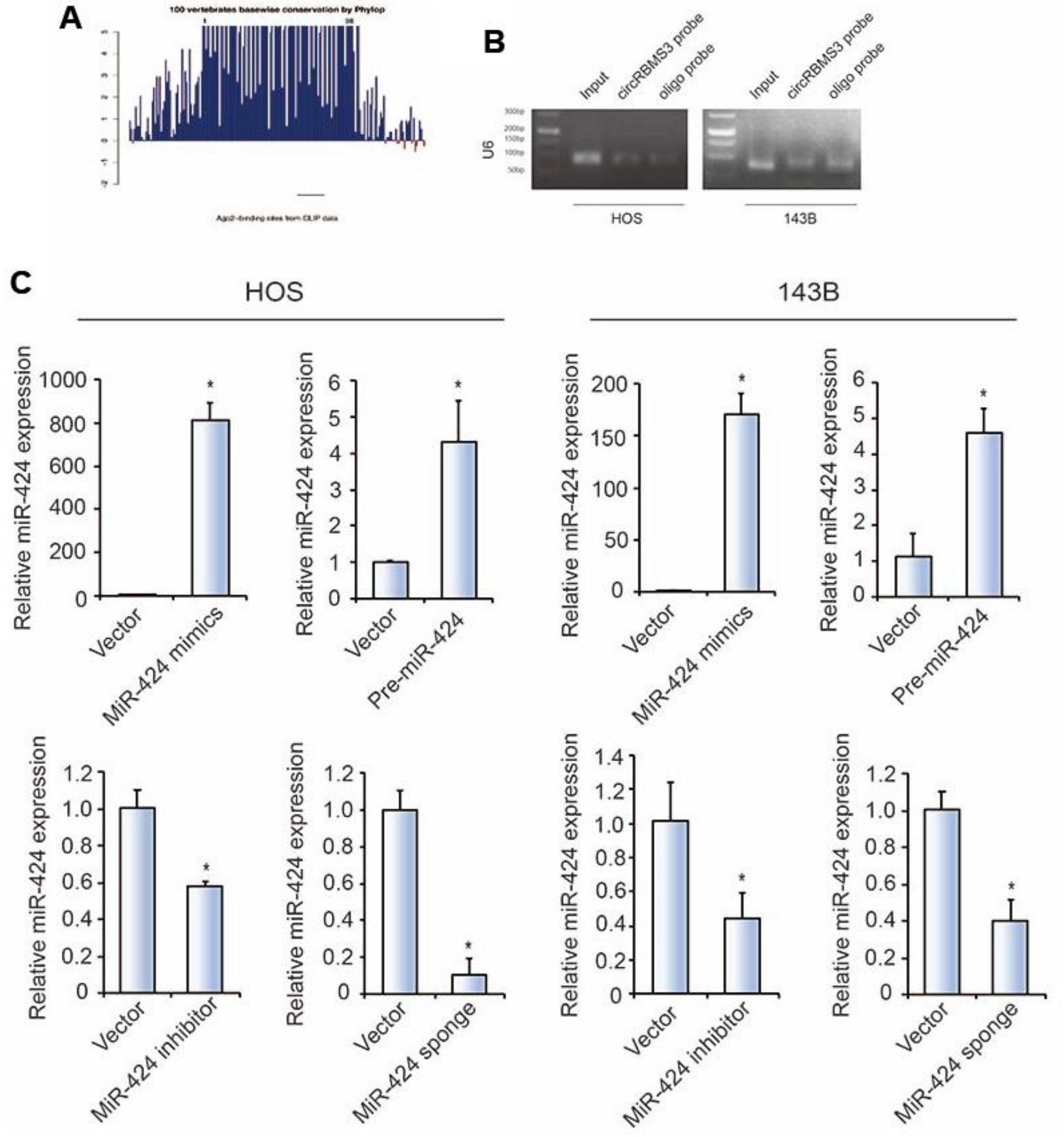

Supplementary Figure 3. Efficiency of miR-424 overexpression and knockdown in OS cells. (**A**) Schematic illustration showing conservation across 100 vertebrate species and *AGO2* binding sites in the circRBMS3 genomic region. (**B**) Lysates prepared from HOS and 143B cells were subject to RNA pull-down assay and tested by qPCR. Relative levels of U6 were normalized to input. (**C**) Efficiency of miR-424 overexpression (miR-424 mimics or pre-miR-424) and knockdown (miR-424 inhibitor or miR-424 sponge) in HOS and 143B cells.

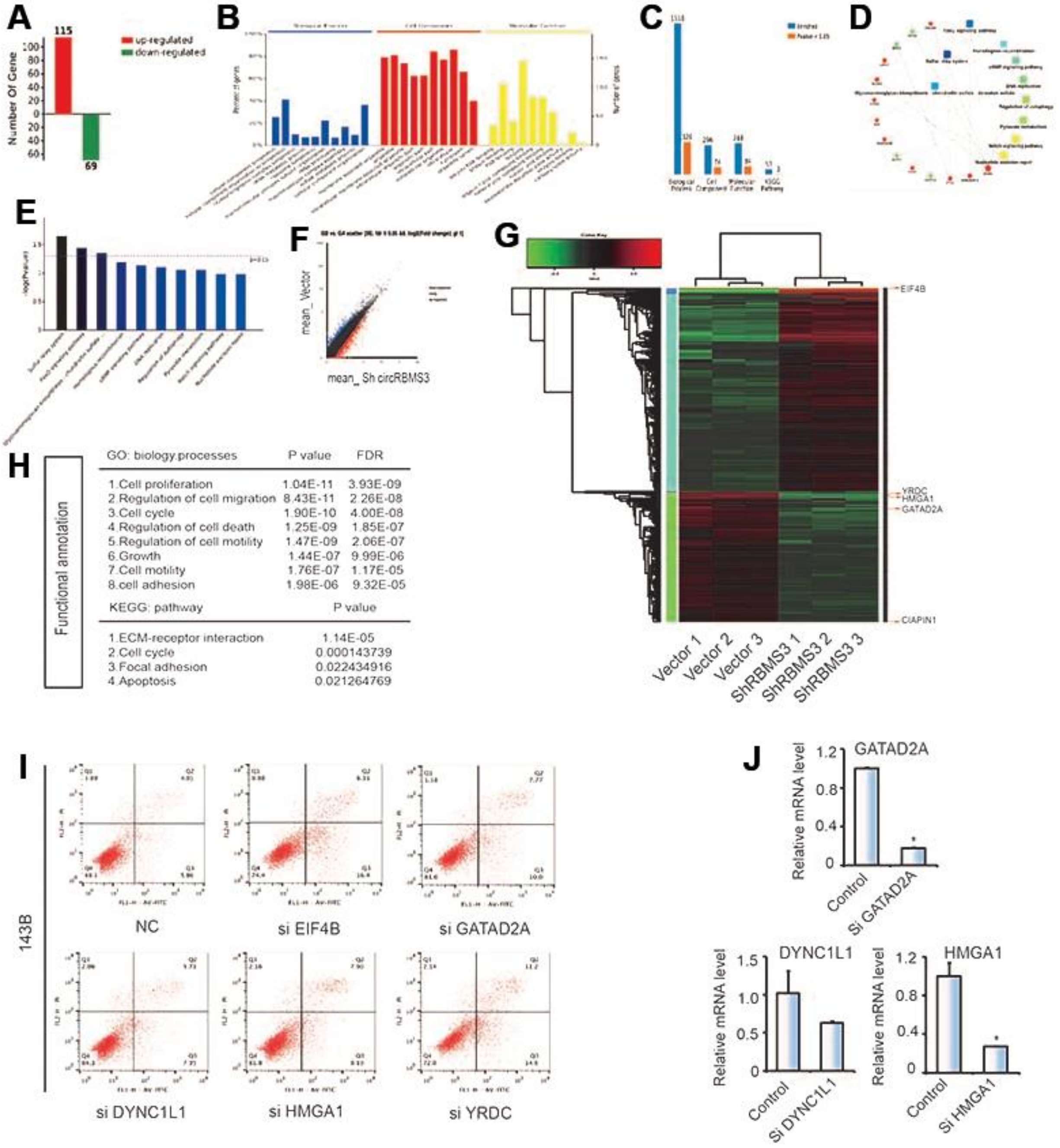

Supplementary Figure 4. (**A**) Expression ratio of 184 differential proteins, 115 up (red) 69 down (green). (**B**) Summary column chart – Gene ontology (GO) enrichment analysis concept map: the Figure shows the first ten of the top of BP, CC, and MF enrichment analysis results. (**C**) Column showing the differences in protein GO enrichment (BP, CC, MF) and KEGG pathway enrichment results, and significant (p-value < 0.05) values are summarized. (**D**) Analysis of protein-protein interaction shows the top ranking KEGG pathways and the interactions between proteins. (**E**) Customized vertical histogram showing the top ranking 10 entries of the biological process enrichment analysis results. (**F**) Volcano plots of RNA sequencing. (**G**) Heatmap of RNA-seq. (**H**) Go and KEGG results of RNA sequencing. (**I**) 143B cells were transfected with siRNA of *EIF4B, GATAD2A, DYNC1L1, HMGA1* and *YRDC* followed by Annexin V-FITC/PI staining. The percentage of apoptotic cells is shown as the mean ± SD from three independent experiments. * $P < 0.05$, significantly different compared with the vector group. (**J**) Efficiency of target gene knockdown by RT-qPCR experiments.
