# Supplementary material for: A novel circular RNA circRBMS3 regulates proliferation and metastasis of osteosarcoma by targeting miR-424-eIF4B/YRDC axis
Source: Aging (Albany NY). 2023 Mar 9;15(5):1564–90. doi: 10.18632/aging.204567 (PMC10042691; doi:10.18632/aging.204567)
Supplement: Supplementary Table 1 [file aging-15-204567-s002.pdf]

# SUPPLEMENTARY TABLE

**Supplementary Table 1. Top 15 differentially expressed circRNAs (upregulated) in osteosarcoma and chondroma.**

| Id | Log2FC(osteosarcoma/chondroma) | Regulate | Significant | Mean_osteosarcoma | Mean_chondroma |
|---|---|---|---|---|---|
| 19:16790795\|16793319 | 4.87446229929571 | Up | Yes | 3.82767979 | 0.03389881 |
| 1:59805630\|59844509 | 4.42274198 | Up | Yes | 14.2568823 | 0.5693945 |
| 3:29910349\|29941246 | 4.13421094 | Up | Yes | 2.38025213 | 0.04124541 |
| 10:32759992\|32762951 | 4.12224656 | Up | Yes | 3.55077865 | 0.10963589 |
| 6:170852689\|170858201 | 3.89178864 | Up | Yes | 12.3500482 | 0.73873722 |
| 17:4186093\|4210418 | 3.8039949 | Up | Yes | 4.99644877 | 0.26488087 |
| 10:74468041\|74475660 | 3.76790046 | Up | Yes | 11.6283293 | 0.7609642 |
| 8:52773405\|52773806 | 3.7547412 | Up | Yes | 43.3253503 | 3.11701884 |
| 6:79770195\|79770535 | 3.64156625 | Up | Yes | 2.88024131 | 0.13879804 |
| 15:31266517\|31269158 | 3.61655163 | Up | Yes | 2.40739166 | 0.10442389 |
| 8:42317414\|42323435 | 3.22589057 | Up | Yes | 1.38553735 | 0.05877925 |
| 6:163876311\|163956157 | 3.20727759 | Up | Yes | 1.76984863 | 0.10245089 |
| 6:170846322\|170858201 | 3.19972312 | Up | Yes | 3.66183094 | 0.30943658 |
| 5:135489281\|135489852 | 3.12372202 | Up | Yes | 1.25361264 | 0.05529604 |
| 4:154547299\|154553990 | 2.9236355 | Up | Yes | 0.93846363 | 0.03686403 |

(Selection for upregulated CircRNAs: log2FC(osteosarcoma/chondroma) >1 and mean chondroma value > 0).
